# Design of a Game-Based Training Environment to Enhance Health Care Professionals’ E–Mental Health Skills: Protocol for a User Requirements Analysis

**DOI:** 10.2196/18815

**Published:** 2021-02-17

**Authors:** Joyce J P A Bierbooms, Wouter R J W Sluis-Thiescheffer, Milou A Feijt, Wijnand A IJsselsteijn, Inge M B Bongers

**Affiliations:** 1 Tranzo, Tilburg School of Social and Behavioral Sciences Tilburg University Tilburg Netherlands; 2 Mental Healthcare Eindhoven Eindhoven Netherlands; 3 Interaction Design ICT Fontys University of Applied Sciences Eindhoven Netherlands; 4 Human Technology Interaction Eindhoven University of Technology Eindhoven Netherlands

**Keywords:** mental health, skill development, eHealth, games, user-centered design

## Abstract

**Background:**

E–mental health (EMH) offers various possibilities for mental health care delivery, with many studies demonstrating its clinical efficacy. However, the uptake of EMH technologies by mental health care professionals remains to be low. One of the reasons for this is the lack of knowledge and skills in using these technologies. Skill enhancement by means of serious gaming has been shown to be effective in other areas but has not yet been applied to the development of EMH skills of mental health care professionals.

**Objective:**

The aim of this paper is to describe a study protocol for the user requirements analysis for the design of a game-based training environment for mental health care professionals to enhance their skills in EMH.

**Methods:**

The user requirements are formulated using three complementary outputs: personas (lively descriptions of potential users), scenarios (situations that require EMH skills), and prerequisites (required technical and organizational conditions). We collected the data using a questionnaire, co-design sessions, and interviews. The questionnaire was used to determine mental health care professionals’ characteristics, attitudes, and skill levels regarding EMH and was distributed among mental health care professionals in the Netherlands. This led to a number of recognizable subuser groups as the basis for personas. Co-design sessions with mental health care professionals resulted in further specification of the personas and an identification of different user scenarios for the game-based training environment. Interviews with mental health care professionals helped to determine the preferences of mental health care professionals regarding training in EMH and the technical and organizational conditions required for the prospective game-based training environment to be used in practice. This combination of requirement elicitation methods allows for a good representation of the target population in terms of both a broad view of user needs (through the large N questionnaire) and an in-depth understanding of specific design requirements (through interviews and co-design).

**Results:**

The questionnaire was filled by 432 respondents; three co-design sessions with mental health care professionals and 17 interviews were conducted. The data have been analyzed, and a full paper on the results is expected to be submitted in the first half of 2021.

**Conclusions:**

To develop an environment that can effectively support professionals’ EMH skill development, it is important to offer training possibilities that address the specific needs of mental health care professionals. The approach described in this protocol incorporates elements that enable the design of a playful training environment that is user driven and flexible and considers the technical and organizational prerequisites that influence its implementation in practice. It describes a protocol that is replicable and provides a methodology for user requirements analyses in other projects and health care areas.

**International Registered Report Identifier (IRRID):**

RR1-10.2196/18815

## Introduction

### Background

Technology can offer significant benefits to mental health care delivery. This includes lowering the threshold to seek help, the possibility of more time- and place-independent health care delivery, and the enhancement of patients’ autonomy [1-3]. Despite the proven efficacy of technology in mental health care [2,3], the uptake of technology among mental health care professionals has remained slow [4-6]. The threshold may even be higher in mental health care than in other health care areas where technology is introduced [7]. This may be because of the focus on the interpersonal relationship between a client and a therapist, where the need to create rapport is high but the perceived possibility to establish this using eHealth remains low [8]. This creates the need to take action to address the causes of these barriers and give room to the potential benefits of technological innovations in mental health care and in other health care areas. Scientific research on the factors that influence the uptake of eHealth reveals that this is dependent on factors such as the characteristics of technological innovation, the internal and external context of the organization, the characteristics of the health care professionals, and the way the implementation process is managed [9]. This also applies to the use of eHealth tools in mental health care, hereafter referred to as e–mental health (EMH) [4,10,11]. One of the most important factors hampering the adoption of EMH by mental health care professionals concerns the lack of knowledge and skills of mental health care professionals in effectively finding and using web-based technologies [8,12-14]. The skills that enhance the ability of health care professionals to effectively use EMH tools include, besides having general digital skills, the use of different communication approaches (eg, compensating for the lack of nonverbal cues and contextual information), choosing the appropriate digital communication channels in each situation, handling boundaries in web-based contact, and knowledge about up-to-date technological possibilities [13,15,16]. In other words, to increase the adoption of EMH, mental health care professionals need to find opportunities to enhance these different skills. To achieve a sense of self-efficacy among mental health care professionals to use EMH, a potential strategy is to offer mental health care professionals training possibilities based on the concept of serious gaming [17].

### Serious Games for Skill Enhancement

Games are usually seen as a leisure activity in which the main aim is to entertain the user. Serious games are (digital) games that are applied for purposes other than entertainment [17-20]. There is usually an educational purpose that is offered in a playful and engaging way [17,19]. Serious games are increasingly used for training in several areas, for example, in aviation, in the military, and in various health care disciplines [20]. The effectiveness of using serious gaming elements in training has been demonstrated in a number of studies [17,21]. One of the most important advantages of using serious gaming elements is that it offers the possibility to learn by gaining new hands-on experiences, instead of merely reading or hearing about it [21,22]. In addition, adding serious gaming elements to regular training methods offers a unique combination of simultaneously educating and engaging users [23]. Furthermore, serious games have multiple learning outcomes (eg, cognitive skills, motor skills, affective learning outcomes, and communicative learning outcomes) that cannot always be gained through more traditional learning methods [24]. Several studies have shown that serious gaming is an effective method for training health care professionals [17]. Examples are simulations to practice surgery, games to practice diagnostic reasoning, and quizzes to practice knowledge about pathology. Wang et al [17] conducted a review that showed that overall serious gaming as a training or learning tool is growing in different health care areas and that most studies included in the review report about this as an effective way for skill development. The characteristics of serious gaming (ie, an engaging form of education, enabling hands-on experiences, engaging, and serving multiple learning outcomes) and the proven effectiveness for training health care professionals are strong arguments to believe that providing serious game–based training to mental health care professionals can be an effective way to enhance their skills in using EMH. In addition, serious games offer a safe and social environment to develop skills in multiple situations and for multiple purposes that could otherwise be difficult, expensive, or unethical to experiment with in a real therapeutic setting [25,26]. Such training possibilities have not been designed yet specifically for mental health care professionals.

### The Identification of the User Requirements

To address such a design challenge, the first step is to identify the user requirements that such an environment should meet. By addressing these needs, professionals are more likely to engage in and benefit from the game-based training possibilities that are offered [27]. Having a clear picture of user needs regarding the innovation or, in other words, answers to the questions “For whom are we developing this product?” and “Why would someone want to use this specific tool?” is essential for users to eventually adopt an innovation [27]. On the basis of the assumption that real-life situations should be at the forefront of the design process, the interaction between users and designers within this process is crucial to make decisions about the design. Therefore, end users should be actively involved from the beginning [28-32]. Before a first prototype can be designed, it is important to determine the core functionality of the innovation and how it can become a meaningful solution by establishing the perspective of the target user group [33-35].

### Objective

This study protocol describes research to define the user requirements for a serious game–based training environment for mental health care professionals to train their EMH skills. The user requirements analysis resulted in three complementary outputs: personas (lively descriptions of potential users or user groups), scenarios (a detailed description of situations that require EMH skills), and prerequisites (the required technical and organizational conditions). This protocol describes the methods used to deliver these 3 outputs and can be used for similar projects in (mental) health care by providing a detailed description of the procedures in a user requirements analysis.

## Methods

### Study Design

#### Multiple Methods

**Figure 1:**
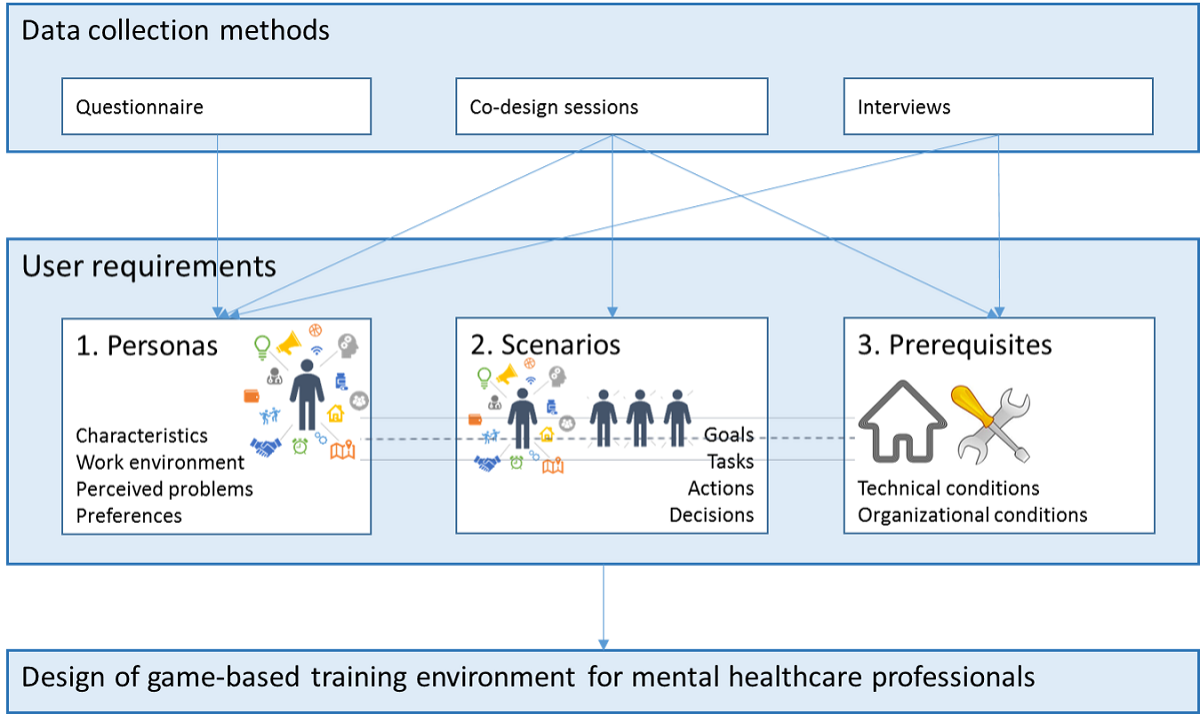
Study design: multi output approach.

This study entails an explorative research design using quantitative and qualitative methods: a questionnaire, co-design sessions, and interviews. The multiple methods approach adopted here incorporates the use of two or more different methods in one study. Unlike a mixed methods design, it does not depend on the integration of data gathered from the different methods, rather provides space to a variety of methodological combinations [36-39]. In this study, a multiple methods design is a more suitable approach as the different methods address different questions, and it would therefore be difficult to truly integrate the data. First, a questionnaire was used to explore the relevant characteristics, work contexts, and practices of mental health care professionals. This includes their attitude and acceptance regarding EMH and respondents’ experienced skill levels to use EMH in their clinical practice. The data provided insights into a wide variety of different and sometimes conflicting user needs. Following this, we grouped user needs to identify a number of different user types within the end user group (mental health care professionals). This provides information about possible differences in user needs regarding a game-based training environment. In addition, mental health care professionals participated in a co-design session to gain a more detailed understanding of the work context of mental health care professionals and the general needs and preferences they have in relation to a game-based training environment. Parallel to this, mental health care professionals were interviewed about specific individual needs regarding a game-based training environment. Subsequently, 2 more co-design sessions were held to develop specific user scenarios for a game-based training environment. This multiple methods approach generates a more in-depth and specific understanding of the requirements, because the data collection identifies different types of end users, with different needs, skill levels, and preferences regarding a game-based training environment (Figure 1).

#### Quantitative and Qualitative Data Collection Methods

The use of both quantitative and qualitative methods for identifying user requirements is grounded in the literature about the development of personas within the context of interaction design [40,41]. In his seminal book *The Inmates Are Running the Asylum*, Alan Cooper [42] introduced the use of personas as a practical design tool. While the Cooper method was originally more qualitative in nature, Pruitt and Grudin [43] used a mix of quantitative and qualitative data to identify and describe user needs. In a later stage, Cooper also added quantitative research to the development of personas [41]. Conducting quantitative research can be seen as an efficient way to assemble as much reliable data as possible [43]. However, in general, there also appear to be many difficulties in the interpretation of this quantitative data for the purpose of distinguishing user groups and truly understanding user needs [42,43]. Therefore, in this study, we combined quantitative and qualitative methods that led to results that are (1) highly representative because of the large and varied number of respondents that can be reached (questionnaire), (2) in depth and specific about the different types of users and their needs in a game-based training environment (interviews), (3) an integration of different user perspectives into a common understanding of the design requirements (co-design session), (4) useful for a number of different scenarios that promote the game-based training environment to fit in the clinical practice of mental health care professionals (co-design sessions), and (5) useful for a specification of the technical and organizational prerequisites that need to be met to foster a successful implementation of the envisioned environment (interviews).

The multiple methods design generates a specification of the requirements in terms of personas, scenarios, and technological and organizational prerequisites (Figure 1), which are commonly used aspects within user requirements specifications [43-47]. In this study, these aspects are used as complementary outputs.

#### Outputs: Personas, Scenarios, and Prerequisites

By developing *personas*, designers try to understand the different types of users and what drives them, in order to truly empathize with them and connect to their needs in the design process [46-48]. Personas are lively and concrete descriptions of potential end users, made by adding attributes to a number of identified subgroups within the potential user group of a product [43,48,49]. In these personas, users’ characteristics and possible goals and needs regarding the achievement of these goals are described and the way in which these differ between the subgroups [43,47-51]. In this study, personas are described by (1) the characteristics of the professionals (age, gender, work experience, and educational level); (2) their work context and practices, including the type of patients they see, the type of interactions these professionals have with their patients, and the situations that they come across that could be supported by using EMH; (3) the perceived potential value and actual adoption of different EMH applications for therapeutic interactions with their patients (attitude) in relation to their current skills regarding the use of these different tools; and (4) their preferences, that is, their ideas about how to acquire these skills and increase their use of EMH. By creating *scenarios*, designers are able to create story lines, related to the personas, for the content development of a game-based training environment [52,53]. These scenarios incorporate details about different situations in which mental health care professionals potentially use EMH. Such scenarios can also be seen as the situation a persona *walks through* [50]. This entails a description of several scenarios that specify the expected benefits of a variety of EMH tools (goals), a step-by-step description of the process (tasks), the execution of these steps (actions), and the decisions mental health care professionals make in these situations. Each scenario ends with a conceptual idea of how a solution can be designed within the game-based training environment that addresses that specific situation. Furthermore, the data collection generated knowledge about the issues that need consideration when transferring from design to implementation in terms of the *technical and organizational prerequisites* that influence users’ (mental health care professionals) intention to actually make use of a game-based training environment. This offers a more holistic perspective on the process by not only focusing on the end user but by also considering what is needed from other stakeholders, such as managers and Information and Communication Technology departments [44,45]. This delivered a set of prerequisites related to the design, content, and facilitation by the organization, which are required to launch the game-based training environment.

### Recruitment

To ensure a good representation of the target user group for each data collection method, professionals from various disciplines that are involved in the direct care delivery process within mental health care were included in the research. The structure of mental health care professions that was officially acknowledged by the Dutch Health Care Authority was used to determine these disciplines, leading to the following clusters of professionals: medical professionals, psychotherapists, psychologists, nursing professionals, social workers, expressive therapists, and paramedical professionals in mental health care. Professionals working in mental health care that are not directly involved in client care (eg, technical support, finance employees, housekeeping) are not likely to use EMH and are therefore not included in this study. The research population that was approached represents the primary users of EMH and therefore the users of the anticipated training environment. This enhances the accessibility of the intended training environment for professionals from multiple disciplines within mental health care with a variety of skill levels and subsequent training needs. In the following sections, the recruitment strategy for each data collection method is specified.

#### Questionnaire

Respondents for the questionnaire were recruited directly at 5 large mental health care organizations in the Netherlands. Furthermore, respondents were recruited through web-based communication platforms of professional associations from different disciplines in mental health care specified earlier. Owing to the explorative nature of this questionnaire, we were not able to execute a statistical power analysis to define a sample size aim. According to Daniel [54], the sample size determination largely depends on the research design. For exploratory research focusing on a single topic and being performed at a national level, which is the case for our questionnaire, a general rule of thumb is that one should have at least 400 respondents [54]. The sample size in our study of 432 respondents complies with the recommendation by Daniel [54]. The responses were collected over a period of 3 weeks for each participating organization. Respondents initially received a message to complete the questionnaire in 2 weeks. After 2 weeks, a reminder was sent to the potential respondents of the 5 participating mental health care organizations, which allowed for 1 week additional response time. The introduction of the questionnaire informed respondents about the purpose of the research project and the protection of their personal data and provided an informed consent button.

#### Co-Design Sessions

To contribute to the description of users and ideas about a game-based training environment from multiple viewpoints, a co-design session was organized involving 9 participants, including mental health care professionals, EMH supporting staff, designers, and researchers. In recruiting participants for this first co-design session, maximum variation purposive sampling was applied using *profession* to maximize the variation. This led to the inclusion of mental health care professionals from a broad range of mental health care disciplines in the sample (eg, psychologists, psychiatrists, nurses, etc) with various levels of adoption of EMH [8] and subsequently a variety of needs regarding knowledge and skill enhancement. Around 6 to 10 participants are considered optimal for a focus group with maximum variation; however, it depends on the context [55]. Although we intend to develop a training environment that is usable for different disciplines in mental health care, we included 9 participants for maximum variation. The participants were recruited at GGzE, a mental health care provider in the southern part of the Netherlands. GGzE is also a partner in this project and a committed stakeholder in the co-design process. The participants were invited by email and were informed about the purpose of the study. When participants agreed to participate, they received more specific information and informed consent forms that they needed to sign when they decided to join. For the 2 co-design sessions that were aimed at developing scenarios for our game-based training environment, we used an expert purposive sampling method in which the same participants took part in each of the sessions to build on the ideas that were expressed earlier. The experts represented the users of the game-based training environment (n=3), game developers (n=3), innovation experts (n=2), and researchers (n=2).

#### Interviews

The interviewees were also selected using a maximum variation purposive sampling strategy and were also recruited at GGzE. We aimed to recruit between 15 and 20 interviewees to guarantee maximum variation in profession and level of adoption of EMH. The interviewees were approached through email. Upon written confirmation of their intended participation, an interview was scheduled. The interview started with a short explanation of the purpose and procedure of the interview and the interviewees were asked if they agreed to the recording of the interview. Upon confirmation, the interviewees were asked to fill in an informed consent form. After 17 interviews, we found that no new data were collected and that data saturation was reached.

### Data Collection Procedures

#### Questionnaire

To determine mental health care professionals’ characteristics, attitude, use, and skill levels on different EMH tools, a questionnaire was developed that uses the current state of the art in EMH and relevant literature on EMH adoption, literacy, and skills [8,12-14]. The questionnaire (Multimedia Appendix 1) started with a general introduction and informed consent. Subsequently, a definition of EMH was given that underlies the different questions that followed. The respondents were then asked to answer 7 questions regarding their adoption of EMH, the type of clients they see, the type of treatments they provide, and the extent to which they think EMH is beneficial to these types of clients and treatments (Multimedia Appendix 1, questions 1-7). These questions were followed by 3 questions where the respondents were asked to self-assess their skills regarding EMH on a scale from 1 to 5 (Multimedia Appendix 1, questions 8-10). General skill levels and specific levels for different types of skills (eg, digital skills, communication skills, etc) were measured. Then the respondents were asked to score 29 statements (Multimedia Appendix 1, question 11) regarding their attitude, beliefs, and perceptions on EMH on a Likert scale from 1 to 5. Finally, 8 general background questions were posed (Multimedia Appendix 1, questions 12-19) with the purpose of gathering descriptive information about the population’s characteristics and their work environments (eg, age, gender, work experience, type of organization, profession).

#### Co-Design Sessions

In the first co-design session (March 2018), the aim was to generate a common understanding about mental health care professionals’ work context and detailed descriptions of the client trajectories that they encounter. Furthermore, the possibilities of applying EMH in this context and their need for knowledge and skill enhancement regarding EMH were identified and discussed. Participants were asked to jointly reflect on these insights and translate this in to ideas for a game-based training environment. This co-design session took 3 hours and demanded no preparation time for the participants. During the session, cards were used to reflect different possible treatment situations, to support participants in *drawing* a client journey. Following this, scoring cards were used on which participants could indicate in which situations EMH tools could be valuable. Finally, the participants were asked to use drawing and crafting materials to visualize their ideal game-based training environment. The 2 co-design sessions that were aimed at developing scenarios (December 2019) took place after analyzing the data from the questionnaire, interviews, and the first co-design session, which means that information about the potential users of the game-based training environment, or the *persona*, was available. In the 2 co-design sessions aimed at scenario development, further elaboration took place regarding story lines that could serve as a basis in a skill-developing game. The session was led by a facilitator, and participants were asked to brainstorm in small groups about different story lines in which a mental health care professional treats a fictional client. Afterward, the different results of the 3 groups were discussed and used by the design team to create 2 story lines. These 2 story lines were refined in a second co-design session in which mental health care practitioners were asked to reflect in detail on the stories that were drawn by the designers.

#### Interviews

The interviews consisted of 2 parts. The first part of the interview was used to gather in-depth information about the specific needs and preferences of mental health care professionals regarding skill enhancement in EMH. This in-depth information was added to the questionnaire data by making more elaborate descriptions of the user groups resulting in personas. These detailed descriptions of the users of the envisioned game-based training environments enable designers to make choices that strongly align with the needs of mental health care professionals. The second part was aimed at identifying the technical and organizational prerequisites to use this environment. The interviews were semistructured to allow for exploring different views on what is needed to develop and implement the envisioned training environment. The topic list was based on the literature on technology acceptance [56] and game-based learning [57,58]. The first questions covered the respondents’ general view on EMH, their current use of EMH, and their experienced skill levels. Subsequently, items were discussed regarding their learning goals on using EMH and how they would perceive a game-based training environment as a tool to enhance their EMH skills. Finally, questions were asked about technical and organizational requirements that should be met to successfully implement such an environment. The topic list was reviewed by another researcher and slightly adapted. After 2 interviews, minor changes were made to the topic list based on the perceived interview flow. The interviews were conducted between May and November 2018.

### Data Analysis

#### Questionnaire

On the basis of the data gathered through the questionnaire, the users could be clustered into a number of subgroups based on shared characteristics that were found in the data. From the literature, we know that there are differences in the perceived drivers and barriers to using innovations [8]. It is therefore important to capture these differences and to understand the important variations that may influence the choices made in developing a game-based training environment. This identifies different possible user groups that may lead to a variance in the solutions offered to serve the target population. Clustering the data is a mode of variance testing that enables us to capture these differences. Clustering can be performed based on a key differentiator that is determined a priori [48-50] or the clusters can flow from the data without appointing specific variables that should determine the clusters [59] or a combination of both in multiple iterations [49,60]. In this study, we used a combination of both approaches in a 2-step analysis: (1) by performing a statistical cluster analysis [61] to identify the number of subgroups in the data set and (2) by performing an analysis using descriptive statistics (eg, frequencies and crosstabs) to identify the detailed characteristics of these user groups and determine whether changes should be made in the initial clustering. In this second iteration, the levels of adoption of EMH [8] were used as a key differentiator to assess the subgroups. Data on the perceived value of EMH and the assessment of different EMH tools, the skill levels, and the type of skills that professionals feel they need to acquire were attributed to the different subgroups. The results of both clustering methods were synthesized, after which the prefinal subgroups were determined based on the variance within the data. Qualitative data forthcoming from the co-design sessions and interviews led to the final clustering solution.

#### Co-Design Sessions

The co-design sessions were recorded using a video camera, microphone, and live note-taking. The recordings and observations were organized and analyzed using thematic coding [62]. Thematic coding is used to cluster qualitative data according to predefined, often theoretically driven, themes by organizing and analyzing the data [62]. It is a useful method to gather information on experiences, viewpoints, attitudes, and social phenomena [62]. In this study, themes were determined based on the purpose of the analysis. For the first co-design session, the purpose was to gather more in-depth information about mental health care professionals’ context and their ideas and perspectives on a game-based training environment for EMH. This resulted in the following main themes: client journey, valued EMH tools, self-assessed skills, perceived learning needs, and game requirements. The second and third co-design sessions were aimed at developing scenarios that led to an approach in which the themes were derived from the elements that constitute a scenario or story line. These elements are the main goal or purpose of a game-based training environment, the tasks and actions that are important for mental health care professionals in their health care delivery, and the situations in which a decision may take place on whether to use EMH. The main themes for each type of co-design session were broken down into a number of elements that contributed to the relevant knowledge (subthemes). The data of the co-design sessions were coded according to these subthemes. Next, based on all different codes, the initial themes were reassessed and combined until all data were accurately attributed to the different themes. The data of the first co-design session were combined with the data of the questionnaire and were processed into the initial user requirements document. This was done by a junior researcher and checked by a senior researcher. The results of the second and third co-design sessions were used to further specify specific game requirements and to decide on the type of solution and content. This part of the data analysis was performed in cooperation between developers and researchers.

#### Interviews

The interviews were recorded (only with explicit permission from the respondent) and were transcribed verbatim. The transcripts were then coded using a thematic coding method and appropriate software for qualitative data analysis [62]. This is in congruence with the overall hybrid research design and with the analysis method that was applied for the co-design sessions. The analysis complemented the needs and requirements analysis by finding users’ perspectives regarding a game-based training environment. Established theory [57,58] was used to define the scope and themes, and at the same time, full space was given to the participants to add to this theoretical knowledge from a more practice-based viewpoint. Thematic coding consisted of a first round of open coding in which the main themes were derived from the data using an open coding approach. These main themes were technical requirements, social requirements, personal factors, managerial requirements, and game requirements. Following the first round, a second coding round was used to find subthemes within the main themes. Examples of subthemes are *perceived ease of use* (technical requirements) and *affinity with computers* (personal factors). The analysis in different coding rounds was conducted by a junior researcher, after which the codes were checked by a senior researcher.

#### Outputs

The data analysis resulted in 3 outputs: personas, scenarios, and prerequisites. These were used to describe the user requirements of the game-based training environment in the design documents. Personas were developed based on data from all 3 sources, scenarios were developed based on co-design sessions, and the prerequisites were based on data from the interviews.

## Results

The research protocol was approved by the Ethical Review Board of Tilburg University in April 2018. At the time of writing this paper, the questionnaire was distributed, 432 people had responded, and the results were available for the design process. A co-design session was conducted to define specific contextual information (n=9) and 2 co-design sessions (n=10) were held to develop the scenarios. The interviews (n=17) were conducted, and the data were analyzed. We aim to report these findings elaborately in a scientific paper describing the results of the user requirements analysis in 2021. The unpublished results are currently used to inform the design process of a game-based training environment.

## Discussion

### Main Discussion of the Protocol

Digital tools are currently underused in mental health care. This is because, in part, of a lack of knowledge, skills, and sense of self-efficacy of mental health care professionals when engaging with a wide variety of available EMH tools. To address this challenge, we are in the process of developing a game-based training environment that offers safe and engaging ways to explore digital tools and become more proficient at their use.

This protocol describes the research and design methods and processes aimed at developing the requirements for such a game-based training environment. This study protocol contributes to an understanding of how user requirements analysis for a game-based training environment in mental health care can be carried out using synthesized expertise from social sciences, clinical practice, and design sciences. To establish this study protocol, researchers from these different disciplines are needed to closely interact to create common ground about the purpose and approach of the project. Knowledge about design thinking [27-29] yields valuable insight into design processes and how to perform research in support of such design processes (eg, a user requirements analysis). The perspectives of social sciences and clinical practice are combined to generate insight into human attitudes and behaviors when confronted with the introduction of technology in mental health care. This resulted in an approach that combines these perspectives to identify user requirements.

While a user requirements analysis in itself is more common in design research, in this protocol, it describes how to approach such an analysis in a health care area where such approaches are rather novel. This has led to a more specific approach that also reflects on the elaborate interactions between researchers in different areas. Although this protocol describes a user requirements analysis for mental health care, it may also be very useful in other projects in mental health care and in health care areas where similar research and design questions may be at hand.

This protocol is particularly valuable because of its holistic approach, in which complementary outputs are proposed. In this multioutput approach, the development of personas and scenarios play an important role in describing users’ needs. More traditional ways of describing user needs are often too generic to fit a variety of potential users of a product [40]. Personas and scenarios allow for differentiation in describing the user group, where distinctive characteristics and situations are used to determine the specific requirements in the design of a product [52,53]. By complementing the personas and scenarios with the technical and organizational preconditions (prerequisites), a more holistic approach is provided to start the design process [45]. The benefits of such a multioutput approach have been discussed in several research papers [43-47]. For example, it is pointed out that personas and scenarios can serve as an interface between the design model and the user model, providing the design team information about users’ goals, skills, and needs and the specific context (ie, treatment situations and organizational context) they are operating in [43-47].

Furthermore, this line of thinking can support multidisciplinary teams in understanding user needs early on in the design process [28,29,31]. Another benefit is the articulation of the *why* of a product in an environment where design thinking is not commonly applied [46]. Owing to the multidisciplinary nature of the research project, we hope that our effort to describe the research approach will contribute to the credibility and reproducibility of this kind of research approach, particularly in health care settings [63-65]. With this protocol, we also aim to contribute to the production transparency and analytic transparency in qualitative research, particularly in design research. To this end, we have transparently reported the aim, methods, and procedures that are used, thereby improving the possibility for the research community to scrutinize the research [63-65] and adopt relevant methods and procedures within their own qualitative research setting. Besides the research community, this particular research project itself benefits from this protocol, as it has added to the common understanding of the purpose of the project and to a shared research approach.

### Conclusions

The envisioned game-based training environment offers an approach that is simultaneously safe, challenging, and engaging. It particularly aims to enhance a number of relevant 21st-century skills that mental health care professionals increasingly need, in addition to their clinical skill set: media and technology literacy, web-based communication skills, flexibility, collaboration, and technology self-efficacy. To develop an environment that can lead to a significant improvement in professionals’ EMH skills, it is important to (1) address real-world issues that mental health care professionals experience and (2) offer training possibilities that address their specific (individual) needs. The approach described in this protocol incorporates elements that enable the design of a product that satisfies these needs. It is user driven and flexible; at the same time, it considers contextual factors that influence its implementation in practice.

## Appendix

Multimedia Appendix 1 Questionnaire (Dutch).

## Abbreviations

EMH: e–mental health
