# Supplementary material for: Design of a Game-Based Training Environment to Enhance Health Care Professionals’ E–Mental Health Skills: Protocol for a User Requirements Analysis
Source: JMIR Res Protoc. 2021 Feb 17;10(2):e18815. doi: 10.2196/18815 (PMC7929747; doi:10.2196/18815)
Supplement: Multimedia Appendix 1 [file resprot_v10i2e18815_app1.docx]

Vragenlijst (questionnaire)

**Part I Introductie (introduction)**

Hartelijk dank voor uw deelname aan dit onderzoek naar eHealth onder zorgverleners. Deze vragenlijst is onderdeel van het door NWO gefinancierde project ‘Serious Games for Professional Skills’ en wordt uitgevoerd door de onderzoekgroep Human-Technology Interaction, Technische Universiteit Eindhoven en Tranzo, Tilburg University, vanuit de Academische Werkplaats "Technological and Social Innovation in Mental Health" waar beide universiteiten bij zijn aangesloten.

***Procedure (procedure)***

Het onderzoek bestaat uit een vragenlijst en duurt ongeveer 15 minuten. Uw deelname is geheel vrijwillig. U kunt zonder opgaaf van redenen weigeren mee te doen aan het onderzoek en uw deelname op welk moment dan ook afbreken door de browser af te sluiten.  Ook kunt u nog achteraf weigeren dat uw gegevens voor het onderzoek mogen worden gebruikt. Dit alles blijft te allen tijde zonder nadelige gevolgen. Daarnaast heeft u als participant een aantal rechten waar u zich te allen tijde op mag beroepen. Deze kunt u vinden in het privacy statement van Tilburg University: http://www.tilburguniversity.edu/nl/disclaimer/privacy-statement/

***Omgang met uw gegevens (data management)***

Bij alle onderzoeken van Human Technology Interaction wordt gewerkt volgens de ethische code van het NIP (Nederlands Instituut voor Psychologen). Wij delen geen persoonlijke informatie over u met mensen buiten het onderzoeksteam. De informatie die we met dit onderzoeksproject verzamelen wordt gebruikt voor het schrijven van wetenschappelijke publicaties en wordt slechts op groepsniveau gerapporteerd. Alles gebeurt geheel anoniem en niets kan naar u teruggevoerd worden. Alleen de onderzoekers kennen uw identiteit en die informatie wordt zorgvuldig afgesloten bewaard gedurende 15 jaar.

***Cadeaubon verloting (gift card lotery)***
Onder de deelnemers worden 10 bol.com bonnen ter waarde van €50 verloot. Mocht u hier kans op willen maken, dan kunt u aan het einde van de survey uw gegevens invullen.

***Vragen (questions)***

Voor vragen of opmerkingen kunt u contact opnemen met één van de onderzoeksleiders, Milou Feijt, MSc (m.a.feijt@tue.nl) of dr. Joyce Bierbooms (j.j.p.a.bierbooms@tilburguniversity.edu), of met de supervisor Prof. Dr. W.A. IJsselsteijn (w.a.ijsselsteijn@tue.nl).

***Instemming onderzoeksdeelname (informed consent)***

Door op de startknop te drukken geeft u aan dat u dit document en de werkwijze hebt begrepen en dat u ermee instemt om vrijwillig deel te nemen aan dit onderzoek van de onderzoeksgroep Human-Technology Interaction, Technische Universiteit Eindhoven en Tranzo, Tilburg University. U gaat ermee akkoord dat wij de gegevens uit deze vragenlijst gebruiken voor de studie binnen het project project ‘Serious Games for Professional Skills’. Tevens geeft u toestemming voor het gebruik van de data en resultaten voor vervolgonderzoek op het gebied van technische en sociale innovaties binnen de geestelijke gezondheidszorg. Mocht u hier bezwaar tegen hebben dan kunt u dit kenbaar maken door een e-mail te sturen naar dr. Joyce Bierbooms (j.j.p.a.bierbooms@tilburguniversity.edu) of Milou Feijt, MSc (m.a.feijt@tue.nl).

**Part II Definitie van eMental Health (definition of eMental Health)**

In deze vragenlijst wordt de term eHealth gebruikt voor alle interacties tussen zorgverlener en cliënt waarbij gebruik wordt gemaakt van een technologie, bijvoorbeeld mail, Whatsapp, Skype, online behandelplatform, games, smartphone apps (bijvoorbeeld voor zelfmonitoring/dagboek) en Virtual/Augmented Reality.

**Part III Questionnaire**

***Adoptie van eMental Health (adoption of EMH)***

1 Welke omschrijving vindt u het beste bij uzelf passen als het gaat om eHealth?

- Ik zit er niet op te wachten om eHealth te gaan gebruiken. (1)
- Het gebruiken van eHealth is geen onderdeel van mijn dagelijkse routine (2)
- Ik gebruik wat voorhanden is. (3)
- Ik ben op zoek naar nieuwe mogelijkheden om eHealth te gebruiken. (4)
- Ik initieer projecten voor de verdere ontwikkeling van eHealth. (5)

| 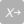 |
| --- |

2 In welke mate denkt u dat de inzet van onderstaande eHealth toepassingen van waarde (kunnen) zijn in het contact met uw cliënt?

Gedeselecteerde keuzes overbrengen van "In welke mate denkt u dat de inzet van onderstaande eHealth toepassingen van waarde (kunnen) zijn in het contact met uw cliënt?"

|  |
| --- |

*Translation: Selected choices of question 2 are transferred and the respondents are asked to indicate their actual use of such EMH tools.*

3 Hoe vaak maakt u gemiddeld genomen gebruik van deze typen eHealth ten behoeve van de behandeling van uw cliënt?

|  |
| --- |

4. Met wat voor soort klachten komen de meeste cliënten bij u? (max. 3 antwoorden mogelijk)

- Stemmingsklachten
- Angst
- Stress/burn-out klachten
- Verslavingsproblemen
- Persoonlijkheids-/ gedragsproblematiek
- Psychose gerelateerde klachten
- Slaapproblemen
- Pijn
- Vermoeidheid
- Seksuele problemen
- Problemen rondom de coping van (chronische) somatische conditie
- Sociale problemen
- Anders, namelijk: .................

|  |
| --- |

5 Wat voor soort behandeling biedt u uw cliënten voornamelijk? (meerdere antwoorden mogelijk)

- Signalering
- Probleemverheldering en diagnostiek
- Psycho-educatie
- Hulp bij zelfmanagement
- Interventie/behandeling
- Terugvalpreventie
- Anders, namelijk: ................. ________________________________________________

6 Maakt u gebruik van eHealth bij de behandeling van deze klachten en zo ja, welk type eHealth gebruikt u het meest?

|  |  |
| --- | --- |
| Stemmingsklachten (x1) | ▼ Geen (5) ... Informatieve website (xx16) |
| Angst (x4) | ▼ Geen (5) ... Informatieve website (xx16) |
| Stress/burn-out klachten (x5) | ▼ Geen (5) ... Informatieve website (xx16) |
| Verslavingsproblemen (x6) | ▼ Geen (5) ... Informatieve website (xx16) |
| Persoonlijkheids-/ gedragsproblematiek (x15) | ▼ Geen (5) ... Informatieve website (xx16) |
| Psychose gerelateerde klachten (x14) | ▼ Geen (5) ... Informatieve website (xx16) |
| Slaapproblemen (x7) | ▼ Geen (5) ... Informatieve website (xx16) |
| Pijn (x8) | ▼ Geen (5) ... Informatieve website (xx16) |
| Vermoeidheid (x9) | ▼ Geen (5) ... Informatieve website (xx16) |
| Seksuele problemen (x10) | ▼ Geen (5) ... Informatieve website (xx16) |
| Problemen rondom de coping van (chronische) somatische conditie (x11) | ▼ Geen (5) ... Informatieve website (xx16) |
| Sociale problemen (x12) | ▼ Geen (5) ... Informatieve website (xx16) |
| Anders, namelijk: ................. (x13) | ▼ Geen (5) ... Informatieve website (xx16) |

7 Maakt u gebruik van eHealth bij dit onderdeel van de behandeling en zo ja, welk type eHealth gebruikt u het meest?

|  |  |
| --- | --- |
| Signalering | ▼ Geen (4) ... Informatieve website (xx16) |
| Probleemverheldering en diagnostiek | ▼ Geen (4) ... Informatieve website (xx16) |
| Psycho-educatie | ▼ Geen (4) ... Informatieve website (xx16) |
| Hulp bij zelfmanagement | ▼ Geen (4) ... Informatieve website (xx16) |
| Interventie/behandeling | ▼ Geen (4) ... Informatieve website (xx16) |
| Terugvalpreventie | ▼ Geen (4) ... Informatieve website (xx16) |
| Anders, namelijk: ................. | ▼ Geen (4) ... Informatieve website (xx16) |

***eMental Health vaardigheden (EMH skills)***

8 Kunt u aangeven hoe vaardig u`zich **over het algemeen** voelt als het gaat om het inzetten van eHealth toepassingen? Kruis aan welke van onderstaande omschrijvingen u het beste bij uzelf vindt passen.

- Ik weet onvoldoende wat er aan eHealth toepassingen beschikbaar is. (1)
- Ik heb een beeld van wat er aan eHealth toepassingen is, maar ik weet niet hoe ik deze moet gebruiken. (2)
- Ik weet hoe ik bepaalde eHealth toepassingen moet gebruiken en hoe ik de basale functionaliteiten kan inzetten. (3)
- Ik ben in staat om zelf te ontdekken hoe applicaties werken en kan daardoor met verschillende eHealth toepassingen goed uit de voeten. (4)
- Ik ben zeer goed thuis in verschillende eHealth toepassingen en ben in staat mijn expertise op dit gebied aan anderen over te dragen. (5)

9 Hieronder volgt een aantal stellingen die gaan over uw vaardigheden ten aanzien van het gebruik van **verschillende typen van eHealth**.  Geef per stelling aan in hoeverre u deze op uzelf van toepassing vindt.


10 Hieronder volgt een aantal stellingen die gaan over **verschillende typen vaardigheden** die een rol spelen bij het inzetten van eHealth in het contact met de cliënt. Geef per stelling aan in hoeverre u deze op uzelf van toepassing vindt.

***Statements over attitudes, opvattingen en percepties van eMental Health (statements on attitude, beliefs, perceptions on EMH)***

11 Hieronder volgen een aantal stellingen over uw houding ten opzichte van eHealth in uw werk als zorgverlener. Geef per stelling aan in hoeverre u deze op uzelf van toepassing vindt.

|  | Zeer mee oneens (1) | Mee oneens (2) | Neutraal (3) | Mee eens (4) | Zeer mee eens (5) |
| --- | --- | --- | --- | --- | --- |
| eHealth sluit goed aan bij mijn werk als zorgverlener. (11_1) |  |  |  |  |  |
| Contact tussen zorgverlener en cliënt hoort altijd face to face te zijn. (11_2) |  |  |  |  |  |
| Ik verwacht dat eHealth voordelen biedt voor de zorg die ik verleen. (11_3) |  |  |  |  |  |
| Ik houd me bezig met het opzetten van initiatieven voor de ontwikkeling van nieuwe eHealth toepassingen. (11_4) |  |  |  |  |  |
| eHealth biedt geen verbetering aan de zorg die ik verleen (11_5) |  |  |  |  |  |
| Vergeleken met collega's maak ik veel gebruik van eHealth (11_6) |  |  |  |  |  |
| Ik ben terughoudend in het inzetten van eHealth (11_7) |  |  |  |  |  |
| Ik gebruik eHealth toepassingen die gemakkelijk zijn in het gebruik. (11_8) |  |  |  |  |  |
| eHealth past niet bij het beroep van zorgverlener (11_9) |  |  |  |  |  |
| Ten opzichte van collega’s neem ik veel initiatief op het gebied van eHealth (11_10) |  |  |  |  |  |
| Ik bezit vaardigheden die nodig zijn om eHealth toe te passen in mijn werk. (11_11) |  |  |  |  |  |
| Ik weet niet hoe ik eHealth in kan passen in de zorg die ik verleen (11_12) |  |  |  |  |  |
| Ik maak het liefst zo min mogelijk gebruik van de computer en andere nieuwe technologieën. (11_13) |  |  |  |  |  |
| Het gebruiken van eHealth toepassingen gaat me gemakkelijk af. (11_14) |  |  |  |  |  |
| Om eHealth te gaan gebruiken moet ik nieuwe vaardigheden leren (11_15) |  |  |  |  |  |
| Het gebruiken van eHealth in mijn werk is voor mij een gewoonte. (11_16) |  |  |  |  |  |
| eHealth heeft geen toegevoegde waarde voor mijn werk als zorgverlener (11_17) |  |  |  |  |  |
| eHealth zou op een grotere schaal ingezet moeten worden. (11_18) |  |  |  |  |  |
| Het gebruiken van eHealth zit niet in mijn systeem (11_19) |  |  |  |  |  |
| Ik heb ervaren dat eHealth de kwaliteit van de zorg die ik verleen ten goede komt. (11_20) |  |  |  |  |  |
| eHealth is een onmisbaar onderdeel van het werk van een zorgverlener (11_21) |  |  |  |  |  |
| Ik heb ideeën over wat er nog meer ontwikkeld zou kunnen worden aan eHealth toepassingen (bijv. toepassing virtual reality, gaming, biofeedback) (11_22) |  |  |  |  |  |
| In de dagelijkse praktijk is het geen automatisme om eHealth in te zetten in de zorg die ik verleen (11_23) |  |  |  |  |  |
| Ik gebruik eHealth toepassingen die eenvoudig voor handen zijn (11_24) |  |  |  |  |  |
| Ik heb geen ervaring met het inzetten van eHealth. (11_25) |  |  |  |  |  |
| In mijn werk probeer ik collega’s aan te sporen eHealth te gebruiken. (11_26) |  |  |  |  |  |
| eHealth heeft voor mij een lage prioriteit (11_27) |  |  |  |  |  |
| Ik maak dagelijks gebruik van eHealth in mijn werk (11_28) |  |  |  |  |  |
| Ik ben geïnteresseerd in nieuwe ontwikkelingen en toepassingen om eHealth te gebruiken in mijn werk als zorgverlener (11_29) |  |  |  |  |  |

***Achtergrondvragen (background questions)***

12 Wat is uw geslacht?

- Man (1)
- Vrouw (2)

| 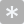 |
| --- |

13 Wat is uw leeftijd?

________________________________________________________________

14 Wat is uw hoogste voltooide opleiding?

- Basisonderwijs
- Voortgezet onderwijs (mavo/havo/vwo)
- Middelbaar beroepsonderwijs (mbo)
- Hoger beroepsonderwijs (hbo)
- Wetenschappelijk onderwijs (wo)

15 In wat voor organisatie bent u werkzaam?

- Basis-GGZ (1)
- Gespecialiseerde GGZ (4)
- Kind & Jeugd GGZ (11)
- Huisartsenpraktijk (5)
- Vrijgevestigde praktijk (6)
- Verslavingszorg (7)
- Algemeen of academisch ziekenhuis (8)
- Forensische instelling (9)
- Anders, namelijk (10) ________________________________________________

16 Binnen welke beroepsgroep valt uw functie? (in geval van meerdere functies, degene waarin u het grootste deel van de tijd werkzaam bent)

- Medische beroepen (bijv, psychiater, arts)
- Psychotherapeutische beroepen (psychotherapeut)
- Agogische beroepen (bijv. maatschappelijk werkende, sociaal pedagogisch hulpverlener)
- Psychologische beroepen (bijv. basis-, kinder & jeugd-, GZ-psycholoog)
- Psychologische beroepen - specialisme (bijv. klinisch (neuro)psycholoog)
- Vaktherapeutische beroepen (bijv. psychomotorisch therapeut, creatief therapeut)
- Verpleegkundige beroepen (bijv. verpleegkundige, sociaal psychiatrisch verpleegkundige)
- Somatische beroepen (bijv. dietist, fysiotherapeut, neuroloog, huisarts)
- Ondersteunende beroepen (bijv. casemanager, psychodiagnostisch medewerker, ervaringsdeskundige)
- Anders, namelijk ________________________________________________

17 Hoe lang bent u al werkzaam als zorgverlener? (in jaren)

________________________________________________________________

18 Bij welke organisatie bent u werkzaam?

- GGzE
- Dimence Groep
- Lentis
- Noord-Holland-Noord
- GGZ Oost-Brabant
- GGZ InGeest
- Parnassia
- Altrecht
- Mentaal Beter
- GGZ BreBurg
- Voorzet
- Vrijgevestigde praktijk
- Anders, namelijk...
- Zeg ik liever niet

19 Heeft u opleiding/training gehad om eHealth tools te gebruiken?

- Ja, tijdens mijn basisopleiding
- Ja, tijdens een nascholing en/of specialisatie
- Ja, door hier een cursus voor te volgen
- Ja, op een andere manier, namelijk
- Nee
